# Albumin-based nanoparticles: small, uniform and reproducible

**DOI:** 10.1039/d2na00413e

**Published:** 2023-01-04

**Authors:** Gudrun C. Thalhammer-Thurner, Paul Debbage

**Affiliations:** a Division of Histology and Embryology, Department of Anatomy, Medical University Innsbruck Müllerstrasse 59 6020 Innsbruck Austria Gudrun.Thurner@i-med.ac.at; b Department of Radiology, Medical University of Innsbruck Anichstrasse 35 6020 Innsbruck Austria; c Institute of Pathology, Neuropathology and Molecular Pathology, Medical University of Innsbruck Müllerstraße 44 6020 Innsbruck Austria

## Abstract

Nanomedicine carries the hope of precisely identifying and healing lesion sites *in vivo*. However, the reproducible synthesis of monodisperse protein nanoparticles smaller than 50 nm in diameter and up-scalable to industrial production still poses challenges to researchers. In this report, we describe nanoparticles, so called Absicles, based on an albumin matrix and prepared by a procedure developed by the authors. These Absicles are monodisperse with tunable diameters ranging from 15 nm to 70 nm respectively. They exhibit long term stability against decomposition and aggregation, exceeding many months. The synthesis of Absicles shows exceptionally high reproducibility concerning size, and is simple and cost-effective for up-scaling. Absicles, bearing appropriate targeting groups, bind with high specificity to colon carcinoma tissue *ex vivo*; they present an attractive platform for further development towards drug delivery applications.

## Abstract 2

Simple and up-scalable synthesis method for human serum albumin nanoparticles with narrow size distribution, tunable size range, stable and reproducible.

## Graphical abstract

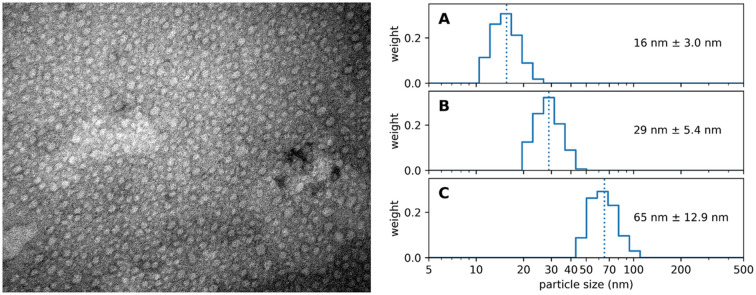

## Introduction

One challenging idea of medicine – already described by Paul Ehrlich,^1^ with his “Chemotherapia Specifica” – is to identify and treat even the smallest malignant lesions in the living body, curing and saving patients' lives. Since the 1970s Nanomedicine has focused on solving this challenge,^2^ creating numerous types of nanoparticles, *e.g.* liposomes, dendrimers, polymers, protein-based particles, carbon-dots, quantum dots, gold particles or ultrasmall particles of iron oxide (USPIOs). However, only a fraction of these entered the clinical market^3–5^ with Doxil® and Abraxane® as the two blockbuster formulations.^6,7^

Abraxane®, in clinical use against metastatic breast cancer, is a 130 nm diameter large co-condensate of Human Serum Albumin (HSA) with paclitaxel, and generates an annual revenue of ∼1 billion US $.^8^ In contrast to most nanoparticles in research which are prepared in highly sophisticated manner, the manufacturing of Doxil® and Abraxane® is surprisingly simple. This is one of the reasons for their success: only simple preparation processes are easily scaled up.^9^ In our development of nanoparticles we heed Prestwich's advice to “embrace complexity, engineer versatility, and deliver simplicity”.^10^

We have made use of this statement by developing nanoparticles in a very simple and reproducible way. They consist of HSA, chosen as matrix molecule because it is one of the longest known and best studied proteins.^11–19^ HSA possesses amino, carboxyl and sulfhydryl groups which provide appropriate structures for attachment of drugs, targeting groups and/or molecules which allow for molecular imaging.

Different methods have been employed to produce protein-based nanoparticles, *e.g.* microemulsion,^20^ desolvation,^21^ sonochemistry,^22^ thermal decomposition,^23^ or the electrospray technique.^24^ The resulting nanoparticles showed interesting properties, however, the difficulties in these methods lie in producing small uniform nanoparticles, in controlling protein precipitation, in intra-batch heterogeneity, in costly and elaborate production, in complex equipment and in limited reproducibility.

The method we present in this report offers a solution to these production challenges: it is simple, does not require the use of organic solvents, and does not lead to precipitation of the protein. In short, monomeric HSA molecules are used for nanoparticle synthesis, the synthesis using only HSA, sodium chloride (NaCl) and glutaraldehyde as chemicals, and heat and stirring speed as physical parameters. These so called Absicles show unique properties: tunable sizes ranging from 15 nm, 30 nm and 70 nm diameter respectively depending on the settings (*e.g.* salt concentration, pH, …), they are monodisperse, non-aggregating even after three-years storage at 4 °C, the synthesis allowing up-scaling and production also in laboratories having only the most basic equipment. Absicles show exceptionally high *ex vivo* stability, are highly reproducible and fulfill all requirements for later theranostic applications.

## Results

### Absicles exhibit spherical shape

**Fig. 1:**
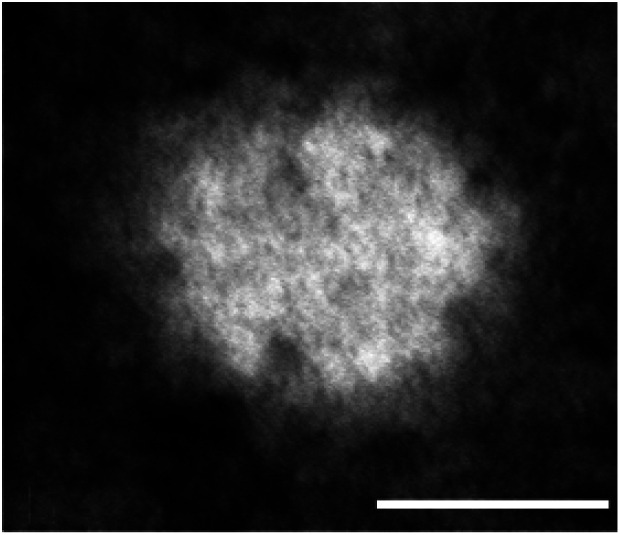
TEM image of a single 30 nm Absicle revealing a rather dense packing of HSA molecules (calibration bar 20 nm, original magnification ×660 000).

Shape is a physical property determining *in vivo* behavior of nanoparticles and sometimes mediating their potential toxicities. Nanoparticles with sharp edges, as *e.g.* carbon nanotubes, can disrupt cellular membranes;^25^ oblate- or rod-shaped nanoparticles show a higher degree of interaction with target cells.^26,27^ Shape also influences the forces which act on a nanoparticle *in vivo*, *i.e.* the shear forces and turbulences in the blood stream. Therefore, in the circulation spherical nanoparticles experience weaker forces of drift and torque than do elongated-shaped particles.^28,29^ It is likely that nanoparticles with sufficient chemical stability and having flexibility allowing them to change their shape slightly can thus enter their target cells intact.^30^ The Absicles presented here are of spherical shape (Fig. 1) and exhibit sufficient stability and flexibility not to be disrupted by *e.g.* diafiltration (see below). The TEM image of Fig. 1 shows a single Absicle of 30 nm diameter, the missing substructure suggests that the HSA molecules within the Absicle are densely packed. The rough surface that we observe could be related to the structure of HSA itself,^12,13^ suggesting the individual HSA molecules are densely packed, or to non-uniform contrasting with uranyl acetate.

### Uniform Absicles with tunable size

**Fig. 2:**
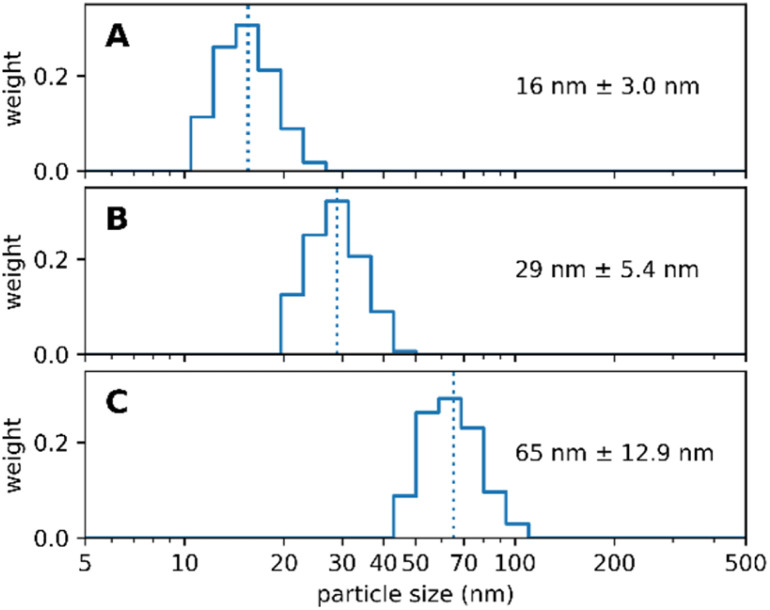
shows three batches of Absicles: (A) of ∼16 nm diameter; (B) of ∼30 nm diameter; (C) of ∼70 nm diameter. Each of the batches revealed a PDI ≤ 1.1.

Wittrup *et al.*^31^ suggested that the size of nanoparticles should be close to the size of the human immunoglobulin G, namely ∼14 nm.^32^ This would offer the best balance between increased circulation time yet still enable the nanoparticle to extravasate the blood lumen towards its target.^33^ As we follow multiple routes for applying our nanoparticles, *e.g.* intravascular or topical, we aim to be able to synthesize nearly any size ranging from 10 nm to 100 nm diameter respectively. It is a crucially important feature of our synthesis protocol that the size of the Absicles is an inherent part of the synthesis, no extrusion as with liposomes or size exclusion chromatography or density gradient technique are required to adjust or filter out undesired sizes. We present here examples for 15 nm, 30 nm and 70 nm diameter, each with a polydispersity index (PDI) of ≤1.1 respectively (Fig. 2); note that in this report a perfectly uniform particle population has PDI = 1.0.

**Fig. 3:**
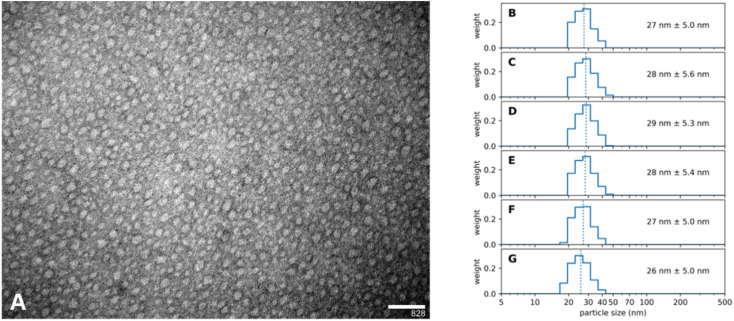
(A) TEM image of ∼25 nm Absicles (calibration bar 100 nm, original magnification ×135 000) (B) to (G) show the volume weighted PCS images of six different batches of Absicles made over a time interval of several months. For the graphical plotting Python software was used applying the original acs.data files generated by the PCS over a run time of 90 minutes per batch. Note the striking intra- and interbatch monodispersity of the Absicles combined with their high reproducibility.

A further outstanding feature of our synthesis protocol is that this synthesis results in a highly standardized inter-batch reproducibility. It was possible to produce a long run of batches over several months, each identical to the others; we show here an example for the 30 nm sized Absicles (Fig. 3). Special care was taken when evaluating PDI values. According to regulatory advice by the European Medical Agency and the Food and Drug Administration of the USA representative size measurements in nanotechnology should always include at least one sizing technique with high resolving power, *e.g.* analytical ultracentrifugation, differential centrifugal sedimentation, nanoparticle tracking analysis or transmission electron microscopy (TEM).^34^ Therefore, we derived PDI values by measuring Absicle diameters of TEM images by hand and then employing a slight adaptation of the methodology used in polymer chemistry as described in Stollenwerk *et al.*^35^ TEM is conducted on dried samples. However, the accordance of the TEM results with the PCS results suggests that the shrinking effect due to drying at room temperature is negligible in our case. Additionally, it should be noted that PCS does not state the actual particle size but the hydrodynamic diameter which means that the actual particle size is a few nanometers smaller. Our data accord with this: TEM indicates Absicle sizes ∼25 nm (Fig. 3A) and PCS shows their hydrodynamic radii as ∼27 nm (Fig. 3B–G).

### Growth kinetics of Absicles

**Fig. 4:**
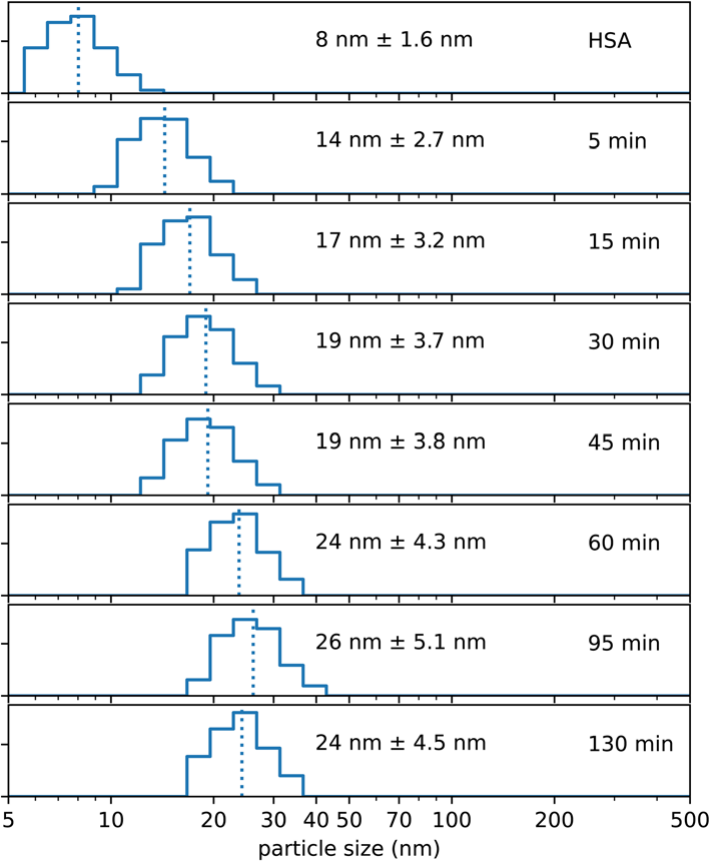
Shows PCS measurements of growth kinetics of ∼30 nm Absicles. The given time points are after glutaraldehyde addition.

Experiments have revealed that the addition of NaCl, the temperature as well as the stirring speed are essential to direct the single HSA molecules into the condition that Absicles can be formed, *i.e.* a condition where the single HSA molecules are positioned very closely to each other due to reduction of the Debye length.^36^ However, the formation of Absicles only occurs after addition of glutaraldehyde, which links the HSA molecules into stable nanoparticles. We made experiments where we measured the increase in size, *i.e.* the development of Absicle formation, after the addition of glutaraldehyde. The time points of the respective PCS measurements were set from 5 min to 130 min after glutaraldehyde addition. Initially we observed a faster growth of the Absicles, which after ∼60 min approached a steady state of about 26 nm diameter (Fig. 4).

### Zeta potential of Absicles

**Fig. 5:**
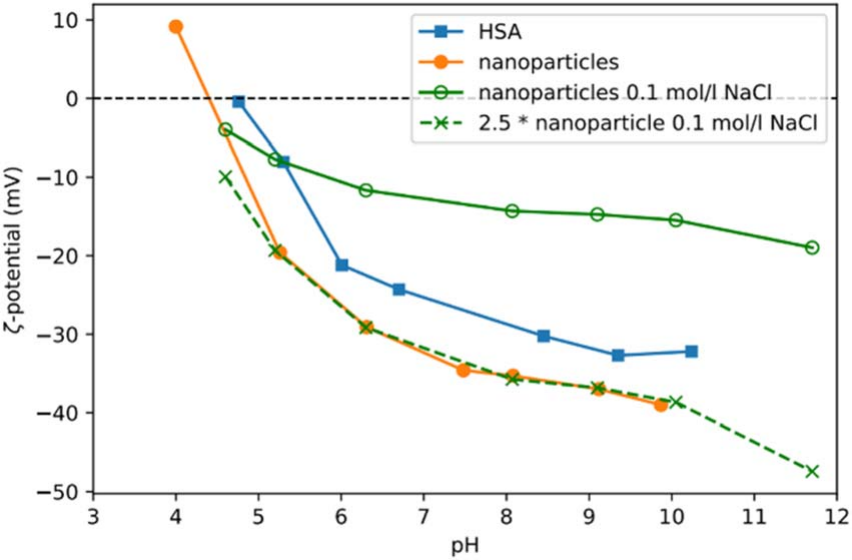
Shows zeta potential measurements of Absicles and pure HSA, for different pH values. Blue line: pure HSA in distilled water; orange line: Absicles after removal of NaCl by diafiltration; continuous green line: Absicles in the presence of 0.1 mol per l NaCl. When scaled by a factor of 2.5 (dashed green line) this data shows a behaviour similar to the Absicles without NaCl.

Storage stability, *in vivo* pharmacokinetics and the potential toxicity are all influenced by the electrical charge of the nanoparticles. Long term storage is facilitated if nanoparticles are charged either positively or negatively thereby reducing their tendency to aggregate due to repulsion effects. However, charged particles show certain *in vivo* behaviours as the cell membrane itself is negatively charged: (i) strongly negatively charged particles may not approach lesion cells closely enough for successful targeting;^37^ (ii) in contrast, positively charged particles can disrupt cellular membranes *in vivo* thereby leading to toxic reactions.^38^ Optimally, nanoparticles for *in vivo* application should exhibit a neutral surface. However, with proteins this is nearly impossible as their biological functions are determined, amongst other things, also by charge. We measured the zeta potential of our Absicles before and after diafiltration at concentrations of 6 to 15 mg ml^−1^ (Fig. 5). We observed that the zeta potential of the Absicles (Fig. 5, orange line) was similar to that of pure HSA (Fig. 5, blue line) but with a slight negative shift of the isoelectric point. This could be explained by the reaction of glutaraldehyde with some of the primary amino groups leading to charge modifications. The presence of electrolytes such as sodium chloride which is used during this synthesis exerts a masking effect over charged areas on the HSA molecules. This results in a reduced zeta potential when measured in the presence of 0.1 mol per l NaCl (Fig. 5, continuous green line).

### Long-term stability against decomposition and aggregation

Stability concerns (i) chemical stability against premature cleavage by enzymes present in the blood and interstitial space enabling the nanoparticles to enter the respective lesion sites functionally intact, (ii) physical stability to tolerate the physical forces present in the blood stream (see above) and (iii) long-term storage stability as a major safety feature in market use.

A means to evaluate the chemical stability of the Absicles was the application of sodium dodecylsulfate polyacrylamide gel-electrophoresis (SDS-PAGE). Theoretically, as the sizes of the Absicles are larger than the pores of the gels being used, Absicles having a high stability should remain at the top of the gel and not enter it. In preparing the Absicles for the gel they were subjected to the denaturing effect of SDS and to heat (95 °C for 5 minutes). Due to these high chemical and physical stresses some break-up was observed, however, they also largely kept their physical integrity as shown by their remaining at the top of the gel. We conclude that the Absicles exhibited a very high stability against physical and chemical stresses, retaining their physical integrity.

**Fig. 6:**
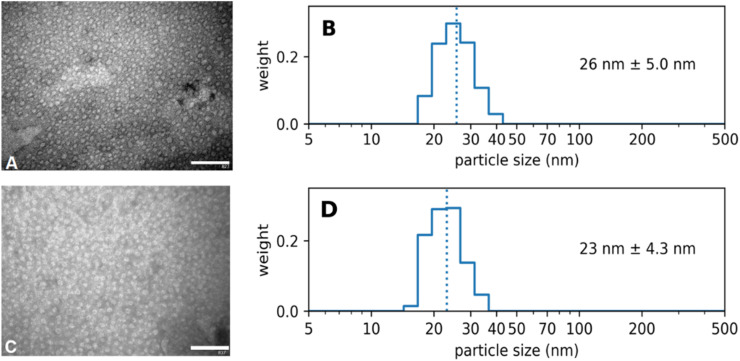
(A) and (B) TEM and PCS images of 30 nm Absicles before diafiltration (calibration bar 200 nm, original magnification ×135 000); (C) and (D) TEM and PCS images of 30 nm Absicles after diafiltration (calibration bar 200 nm, original magnification ×135 000).

Diafiltration is commonly used for purification and can be scaled up to industrial processes.^39^ It was applied for purification but also represented a means to assess the physical stability of the Absicles as diafiltration exerts high mechanical stresses on them imitating the shear forces in the blood stream. The Absicles did not break up during diafiltration (Fig. 6) and only showed a tendency to aggregate in one sample.

**Fig. 7:**
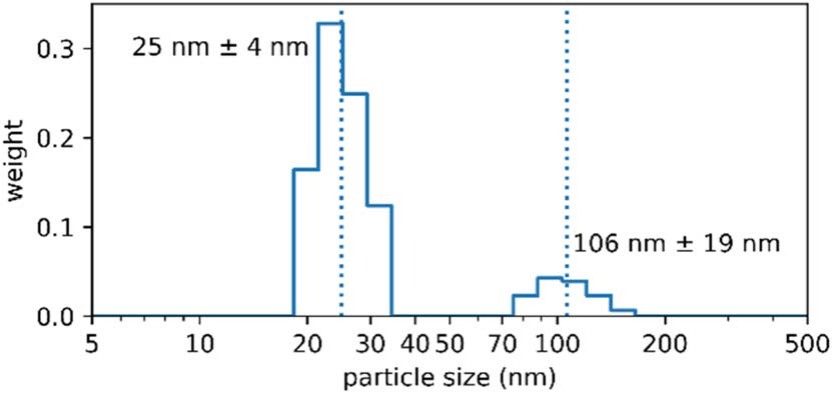
PCS data of a batch of 30 nm Absicles which was produced 3rd March 2017 and since then was stored in a capped glass vial at 4 °C without stirring and without addition of any stabilising reagents. The Absicles show essentially no tendency to aggregate. This image was generated 15th July 2020.

Long-term storage stability, *i.e.* years not weeks, is a major logistical requirement in industry and the market, and is one of the reasons why nanoparticles frequently fail when it comes to translation.^9^ We therefore demonstrated the storage stability of the Absicles by measuring them after three years: they were simply stored in a capped glass vial at 4 °C without stirring and without addition of any stabilizing reagents. The Absicles showed only a minimal tendency to aggregate (Fig. 7).

## Discussion

An ideal nanoparticle for clinical use will be a major work of art: tailored for elegant synthesis, to clinical function, robust, many-sided.

**Fig. 8:**
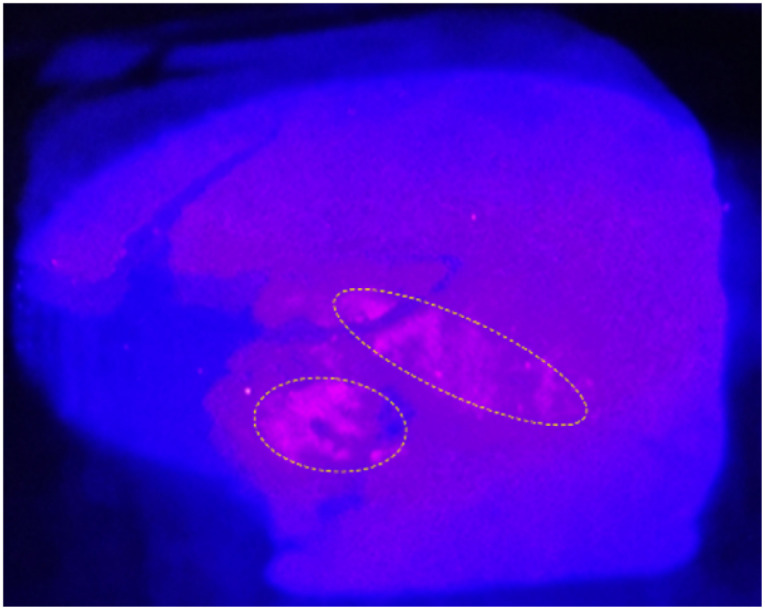
Human colon carcinoma tissue section after incubation with fluorescence-tagged anti-EpCAM-targeted Absicles. The tumour luminal surface is at left. Bright fluorescence appearing in pink localizes specific binding of the Absicles to tumour tissue over-expressing EpCAM (encircled in yellow). Blue excitation light illuminates the entire section. The optical system was adjusted to image both the blue excitation light (intensity <100 mW cm^−2^) and also the emitted red fluorescence. The section contains areas of very strong fluorescence, showing high over-expression of EpCAM. The imaging system resembled that of a human colonoscope; note that the short exposure time required to obtain this image (1/30 s) would allow video imaging in real time.

The synthesis we describe here solves the challenge of creating small, inherently uniform and reproducible protein nanoparticles, which has not been achieved before. These Absicles are especially attractive in so far as they comprise only two molecules, namely HSA and glutaraldehyde. They are made very simply, and show enhanced stability, size and size dispersity standardization. The physicochemical characteristics of the Absicles allow for versatile theranostic applications. They can be produced as 15 nm Absicles for targeting tumours behind blood-tissue barriers, their 70 nm counterparts can be applied for intravascular imaging of *e.g.* atherosclerotic plaques, and the 30 nm Absicles can combine both of the mentioned routes. HSA as matrix molecule bears different functional groups, *i.e.* –COOH, –NH_2_ or –SH,^12^ that are still available after Absicle preparation as we tested by TLC analysis using the respective staining solutions (see Experimental section).^40^ These functional groups can be used to attach imaging or targeting groups as well as different types of drug molecules (Fig. 8). All bonds can be made covalent with built in break-up points increasing their *in vivo* stability until target-docking has succeeded. Generally, analysis of the complete toxicological profile is an essential step do be performed for all nanoparticulate systems before translation into the clinical market. We have begun this analysis for the Absicles, however, this is still an ongoing research. In order to provide insight into the ability of the Absicles to successfully target and image lesion sites *ex vivo* a preliminary experiment was conducted targeting the Absicles with anti-EpCAM antibodies and labelling them with protoporphyrin IX. Despite their negative zeta potential the Absicles were able to target the respective lesion sites without the need to mask their charge by PEGylation or any other means of masking. In Fig. 8 we demonstrate the specific binding of the Absicles to a colon carcinoma.

### Mechanisms for tunable size with small dispersities

Most remarkable was the high reproducibility of the Absicles together with the fact that their size is an inherent part of the synthesis. This unique size standardisation of the Absicles and, therefore later of their functional loadings, underpins and facilitates important safety and regulatory examination of the Absicles, namely the implementation of effective analysis and monitoring methods to analyse their physicochemical characteristics *ex vivo* and *in vivo*.^41^

It became evident that certain aspects of this synthesis must be very robust to produce a series of batches at dates separated by several months and made from different batches of component materials, yet with each Absicle batch being identical to its predecessors.

It is evident that a thermodynamically driven reaction between the interfaces of adjacent HSA molecules precedes the formation of the Absicles. These interfaces are influenced by pH and by the presence of salt and heat. Further, pH and salt strongly influence the charge on molecules such as HSA, and charge obviously seems to play an important role in the formation of the Absicles. Molecules acquire charge when in contact with a polar medium.^42^ Of several possible mechanisms generating this charge, ionization of the surface groups is of particular relevance for proteins: ionization and/or dissociation of carboxylic acid or amino groups, and the net molecular charge determining whether the sign is positive or negative, depend strongly on the pH of the solution. The electric charge *Q* of a protein molecule is the sum of the electric charges of the amino-acids that make it up. The overall net charge of HSA at pH 7 as stated by Peters is −15 elementary charges but is not evenly distributed across the HSA molecule.^12^ In the work reported here, a further indication of the influence of charge was the fact that Absicle size and size dispersity depended strongly on electrostatic interactions. This was shown by varying the pH: at pH 7.0, Absicles of 30 nm diameter and PDI ≤ 1.1 were produced. Varying the pH in either direction altered the size and increased the PDI drastically. This implicates altered charge properties at the protein surface as important for the adhesion of HSA molecules to one another.

A question arises to the mechanism of Absicle formation given the fact that HSA at pH 7 carries 15 negative charges implicating repulsion between single HSA molecules in a solution. How can the molecules approach each other so closely that they can be crosslinked by glutaraldehyde molecules which have a length of only ∼0.7 nm? For a protein molecule with various charge domains at its surface it may not be sufficient that a single (*e.g.* positive) charge domain comes this close to a (*e.g.* negative) charge domain. Close approach is more likely if the charges are masked close to the Stern layer.^43^ The solution to that requirement lies in relatively high salt concentrations (up to 1.6 M NaCl) suppressing electrostatic interactions including repulsion and at pH 7 facilitating the close approach of HSA molecules to one another, and hence their mutual adherence. Although this might indicate a propensity to precipitate out, the experiments showed that this does not occur for sodium chloride up to a concentration of 1.6 M and under the given synthesis conditions. This “ionified” state of HSA is likely to allow sensitive manipulation of the rate at which precipitation occurs.

Flanking experiments have revealed that the concentration of NaCl and therefore the strength of electrostatic interactions are a critical parameter influencing the size of the Absicles. Varying the ionic concentration altered the size of the Absicles produced. This was a strong effect: the change from 1.0 to 1.6 M NaCl altered the resultant Absicle size from 30 nm to 70 nm diameter. Higher ion concentrations evidently mask the charge from charged residues on the HSA surface, thus reducing the Debye–Hückel length^36^ of the charge on the residues and allowing closer approach of the HSA protein molecules to one another. As a result one could expect more opportunities per second for glutaraldehyde to crosslink two HSA molecules and thus a greater rate of production of crosslinked material: the Absicles would “grow faster”. This element of time in the procedure may be of interest for further discussion of Absicle size.

As already mentioned, addition of NaCl neutralizes the protein surface charges, thereby permitting close approach of protein molecules, which would normally lead to protein precipitation. In the synthesis method described here, the stirring speed and, to a lesser extent the temperature, seem to counteract this precipitation. The method presumably involves a complex state of the Gibbs free energy resulting from the numerous electrostatic interactions at the HSA molecule surface. This critical state of the HSA with respect to precipitation results from close proximity to a conformation which minimizes the free energy: the addition of cross-linking molecules as described moves the HSA from close proximity into this conformation, *i.e.* the Absicles. We consider this might be described as quasi-crystallisation leading to aligned, regular structures yielding monodisperse Absicles.

### Mechanisms that form the inner structure of the Absicles

A further and knottier question concerns the high reproducibility of the Absicle sizes, together with the small variation in size within a single batch. Although other protein molecules could be crosslinked by use of aldehydes, the structure of HSA would be predestined for use in creating three-dimensional crosslinked architectures of regular form: two of the natural amino acids forming HSA carry sidechains containing an amino group implicated in crosslinking *via e.g.* glutaraldehyde: lysine (K) (p*K*_a_ = 10.53) and arginine (R) (p*K*_a_ = 12.48). Of these, arginine occurs on HSA only three times at surface-exposed sites (residues 81, 114 and 117) and lysine eight times.^11^ Large stretches of the HSA polypeptide chain assume the form of α-helices in the native conformation, and only relatively short length of chain loop between the α-helices as single chain segments. However, each subdomain of HSA carries one lysine residue within such a single chain segment, precisely at the point where a loop occurs at an exposed site on the surface: residue 174 in domain I, residue 313 in domain II, and residue 444 in domain III.^12^ The charge relationships are difficult to disentangle at these points on the molecule, because the pKa value for *e.g.* lysine can vary from 6.0 to 10.53, depending on the local environment and on the presence of possible proton donors in the close vicinity.^12^ We suggest however, that the presence of lysine at these sites, together with its flexible p*K*_a_ value, renders it a strong potential candidate for acting as carbonyl binding site during crosslinking with glutaraldehyde; none of the other amino acids can be considered strong candidates. The presence of two such lysine residues would permit crosslinking of one HSA molecule with two others to form a chain, but there are in fact three lysine residues and this would allow crosslinking of one HSA molecule with three others, thus forming a lattice. The experimental data presented in this report show that HSA is not crosslinked merely as a lattice within the Absicles, because stacks of lattices would fragment along cleavage planes, as does graphite. The Absicles however do not crumble or flake when exposed to mechanical stresses such as turbulence or passage through a polyacrylamide gel. Evidently the Absicles contain HSA crosslinked to form three-dimensional cage-works. For this, each HSA molecule must contain at least two further carbonyl-binding sites, giving a total of at least five. The five lysines that are required are indeed present at appropriate sites,^11,13^ yet few or no further aldehyde-binding sites are present that would permit formation of a variety of possible architectures. Therefore, the range of possible 3-D architectures in cross-linked HSA may be small. This might be one reason for the highly standardized sizes and size dispersities of the Absicles reported here: there is an optimal crosslinking pattern that appears to be inevitable for the HSA molecule in its native conformation. Consequent to this high standardisation is the form of packing and the packing density of the single HSA molecules within the Absicles. The packing density of such an Absicle is important in order to reveal its drug-loading capacity and its capacity of functionalisation with targeting and/or imaging groups. We consider our TEM imaging data to show a high packing density (Fig. 1).

As the Absicles described in this report exhibited remarkable size homogeneity and were of spheroidal shape, their volume could be readily calculated: *e.g.* an Absicle of 30 nm diameter has a volume of ∼14 137 nm^3^ (volume of a sphere = 4/3 × π × *r*^3^). However, many years of crystallographic studies of albumin from several species, aided by use of the Space Shuttle microgravity environment^11^ culminated in determination of the tertiary structure of HSA as a heart-shaped molecule 8 nm on a side with average thickness of 3 nm, and a calculated molecular volume of about 88 nm^3^.^12,44^ By adding the electric double layer^45^ to an HSA molecule of this volume, one obtains hydrodynamic data consistent with the 100 to 200 nm^3^ derived by these measurements. This means, that by taking the volume of a single HSA molecule to be 88 nm^3^ and assuming a 100% packing density – which is unlikely – as many as 161 HSA molecules could pack into one such Absicle. Inevitably, the shape of HSA, a flexible molecule which twists, bends and flattens under a range of circumstances, to adhere to flat surfaces or to accommodate one or another of its numerous ligands,^46^ is important in packing it into such Absicles.

In addition to shape, electrostatic considerations should be included here as well. As noted above, the positively charged groups at the HSA surface at pH 7.0 are lysine and arginine.^12^ The strong difference in negative and positive charge distribution amongst the three HSA domains provides a rationale to consider that two HSA molecules would pack in a unit cell of the type described by Sugio *et al.*^13^ at almost 180° and with only a small translational shift. We consider this a possible form of packing within the Absicles, and that the mechanisms we have sketched above could result in formation of such a packing. Such packing can indeed reach a very high density. However, biological packing densities in fluids rarely approach the maximum possible values.^47^

**Fig. 9:**
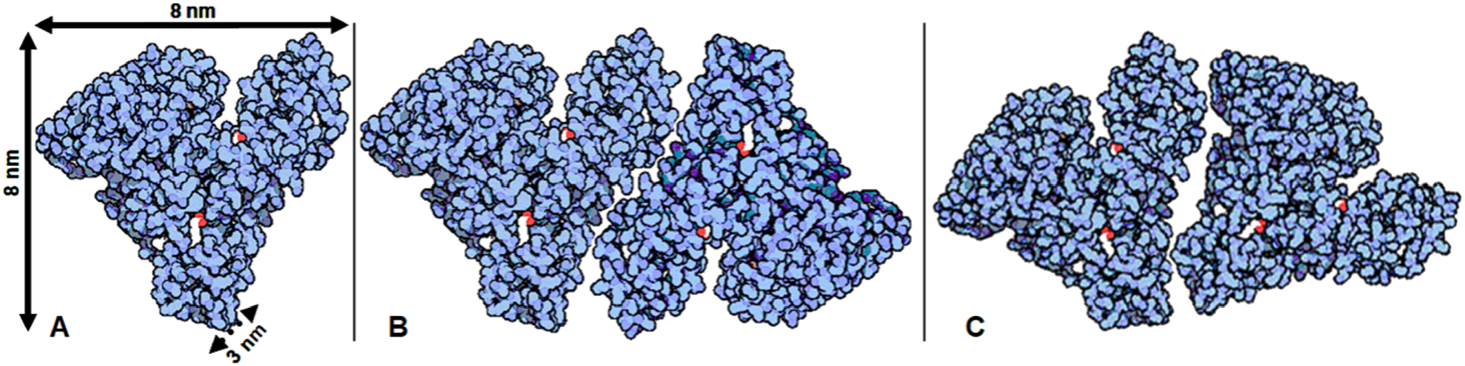
HSA molecules can quasi-tesselate a Euclidean plane, with such planes being stacked to form a solid Absicle. (A) Shows the dimensions of a single HSA molecule as stated by Peters.^12^ The dyads shown in (B) and (C) are two possible fundamental units of such a quasi-tessellation.

Certain assumptions can help with assigning a packing density to HSA molecules in the Absicles. Packing distances must on the one hand be limited by the crosslinking length of the linking structure, *e.g.* glutaraldehyde (∼0.7 nm), on the other hand the single HSA molecules therefore have to approach each other at least as close as 0.7 nm for the crosslinking to occur. The TEM images presented here indicate that the Absicles are spheroidal. Assuming that it is in a static crystalloid state, the HSA in the Absicles is expected to be an oblate asymmetrical spheroid, so it is unlikely to be packed in the same way as spheres would be, and indeed the packing of oblate asymmetrical ellipsoids can reach significantly higher densities than is possible for spheres (which is 0.74), because the ellipsoids can quasi-tessellate a Euclidean plane (Fig. 9), and a number of such planes can in principle be laid over one another to form multi-layer sandwiches. However, the remarkable consistency in the size of the Absicles strongly suggests that one particular pattern of packing is favoured; identifying this pattern would require precise modelling based on the Gibbs free energy.^48^

It remains open to further investigation to confirm this, and to define how numerous such pairs might pack in larger patterns to form the Absicles. The formation of tightly packed, rigid complexes containing dozens or hundreds of HSA molecules can be considered and further investigated in the light of the above discussion. Given that the Absicles are highly standardized, they might be suitable materials for characterization by crystallographic methods.

However, these striking results together with the high reproducibility of equally sized, monodisperse Absicles renders this kind of synthesis a promising way to develop the Absicles further for *in vivo* targeted imaging and drug delivery.

## Experimental

### Materials

Human serum albumin (HSA, fraction V), charcoal, sodium chloride (NaCl), caprylic acid, sodium hydroxide (NaOH), glutaraldehyde 25% (EM grade), sodium cyanoborohydride (NaBH_3_CN), ninhydrin, sodium nitroprusside, bromocresol green, uranyl acetate, concentrated hydrochloric acid (HCl) were purchased from Sigma-Aldrich (Munich, Germany). Rotilabo® syringe filters (PP-Gehäuse, sterile, PVDF 0.2 μm) and Rotilabo® folded paper filters (type 600P, *ø* 185 mm) were purchased from Carl Roth GmbH & Co (Karlsruhe, Germany). The sheets for thin layer chromatography (Silica Gel 60 UV254, Polygram® Sil G/UV254, 0.2 mm; Macherey-Nagel, Germany) were purchased from VWR (Vienna, Austria). Throughout this work, the water used for Absicle synthesis was purified millipore water (Milli-Q system, Millipore, Billerica, MA, USA). Adjustment of pH values was carried out with a pH 211 Microprocessor pH meter, Hanna instruments, ATP Messtechnik GmbH (Ettenheim, Germany), the electrode cleaning solution HI 7061, the electrode storage solution HI 70300 and the pH meter calibration buffers (pH 4, pH 7, pH 10) were purchased from Carl Roth GmbH & Co (Karlsruhe, Germany). Diafiltration was carried out using a Minimate TFF System (Pall Life Sciences, MI, USA). The membranes, Minimate TFF membrane cassettes with Omega® type membranes and cut-off 100 kDa, were purchased from VWR. Electron microscopy measurements were carried out using a Philips CM120 electron microscope. Absicles to be analysed were applied onto formvar-coated copper grids; the grids (150 mesh) and the formvar powder were obtained from Gröpl (Tulln, Austria). Photon Correlation Spectroscopy (PCS) measurements were carried out using a Submicron Particle Sizer NicompTM 380 DLS (Particle Sizing Systems, Santa Barbara, CA, USA). For calibration of the instrument standard 92 nm latex beads (Duke Scientific, Fremont, USA) were used. Zeta Potentials were measured using a ZetaSizer Nano ZSP (Malvern Instruments, Worcestershire, U.K.). SDS polyacrylamide gel-electrophoresis was carried out using the Mini-PROTEAN® Tetra Cell from BioRad (Vienna, Austria). The materials used, namely the gels (Ready Gel® precast gels, 10 well comb, 30 μl load volume), the molecular weight standards, the running and the sample buffers were purchased from BioRad (Vienna, Austria). Substances to prepare the staining and destaining solutions (brilliant blue R 250, glacial acetic acid) were purchased from Carl Roth GmbH & Co (Karlsruhe, Germany) and were prepared manually but according to the protocols given by BioRad. UV measurements were carried out using an Ultrospec III spectrometer (Pharmacia LKB Biochrom Ltd, Cambridge, England).

### Methods

#### Preparation of human serum albumin

HSA in lyophilised form (which is how HSA is commercially distributed) is prone to dimerisation leading to high interbatch variabilities.^49^ Therefore, HSA used in this study was de-fatted and re-conformed^50,51^ before Absicle preparation in order to obtain monomeric molecules in their native conformation. In short, and as described by Abdelmoez *et al.*^47^ according to Chen,^50^ HSA (fraction V) was dissolved in distilled water at room temperature (50 mg ml^−1^, pH 6.7), dry charcoal was added to the solution under continuous stirring (ratio of HSA to charcoal is 2 : 1 w/w) and the pH was adjusted to 3 with 1 M HCl. This suspension was stirred magnetically for 1 hour at 4 °C followed by filtration through a folded paper filter and centrifugation at 5000*g* for 10 minutes. The supernatant was further filtered through a 0.2 μm filter and the concentration was measured at UV280 nm using a spectrophotometer.

#### Preparation of sodium caprylate

To a 10 ml solution of caprylic acid (density *ρ* = 0.91 g ml^−1^) 12.6 ml of 5 M NaOH were added in a 1 : 1 ratio. The resulting precipitate was dissolved by adding 20 ml of a 150 mM NaCl solution leading to a sodium-caprylate concentration of 0.21 g ml^−1^.

#### Re-conformation of human serum albumin

**Fig. 10:**
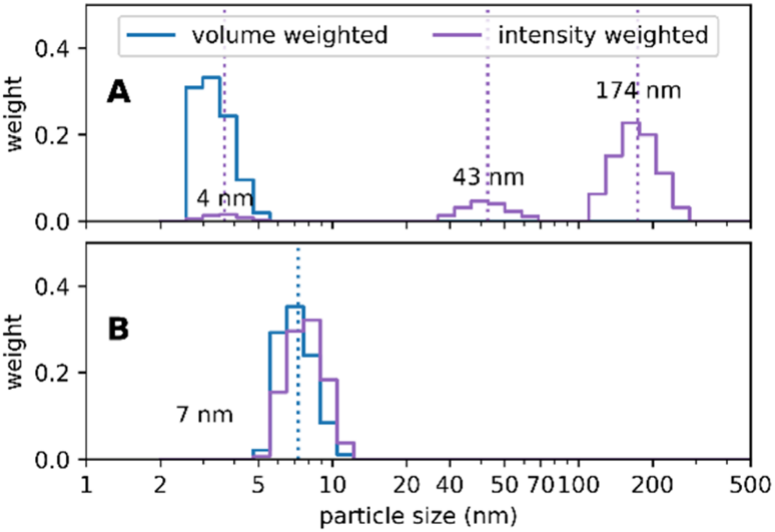
(A) Photon correlation spectroscopy (PCS) data of commercial HSA consisting of small sized molecules of ∼4 nm diameter and aggregates of ∼40 to 170 nm diameter (intensity weighted image, left). These aggregates are not depicted in the volume-weighted image (right) as their overall percentage in the sample is rather low; (B) PCS data of de-fatted and re-conformed HSA revealing monomeric molecules of ∼8 nm diameter.

Re-conformation of HSA was carried out as described by Abdelmoez *et al.*^47^ according to Shrake *et al.*^51^ as follows: the pH of the solution containing de-fatted HSA was adjusted to 4 with 0.1 M NaOH. Then, NaCl, predissolved in distilled water, was added to the solution (calculated to 150 mM for the final volume at pH 7) followed by addition of sodium caprylate (ratio of HSA to sodium caprylate = 30 mg ml^−1^ HSA to 30 mM sodium caprylate). Then the pH was slowly adjusted to 10 by addition of 3 M NaOH. After stirring magnetically for 1 hour at room temperature the pH was adjusted to 7 by addition of 0.1 M HCl and the sample was stirred overnight at room temperature. Fig. 10 shows a comparison of HSA molecules as delivered commercially (Fig. 10A) and re-conformed monomeric HSA molecules ready to be used for Absicle synthesis (Fig. 10B).

#### Synthesis of Absicles

Aqueous solutions of de-fatted and re-conformed HSA with pH 7, protein concentration 10 or 15 mg ml^−1^ in varying sample volumes from 2 to 20 ml were prepared. Then different concentrations of NaCl up to 1.6 M were added in solid form depending on the desired size of the resultant Absicles. The sample was stirred at 400 rpm and at 50 ± 2 °C for 5 hours. Then the sample was cooled down to room temperature (25 to 28 °C). As soon as the sample reached room temperature an aqueous solution of 1% glutaraldehyde was added (ratio of glutaraldehyde to HSA = 1 : 10 v/v). The sample was stirred over night at room temperature. Diafiltration was carried out for purification.

Some of the Absicle batches were further treated with sodium cyanoborohydride (NaBH_3_CN) to reduce the Schiff bases which form between aldehyde functions of glutaraldehyde and the amino groups of HSA. NaBH_3_CN was added at two different ratios to the Absicle samples, either 17.5 mM NaBH_3_CN for 10 mM glutaraldehyde or 175 mM NaBH_3_CN for 10 mM glutaraldehyde.^52^ NaBH_3_CN was predissolved in distilled water and added dropwise to the Absicle samples under continuous stirring. The reaction was completed after stirring for 2 hours at room temperature and diafiltration was carried out for purification. However, no difference was observed between reduced and non-reduced Absicles concerning their physicochemical characteristics, especially their stability. Therefore a reduction step was omitted in later batches of Absicle synthesis.

#### Thin layer chromatography (TLC)

TLC analysis was carried out as described by Abdelmoez *et al.*^47^ as follows: to determine the purity of the starting materials and of the Absicles after diafiltration revealing that no unbound glutaraldehyde remained, samples were spotted onto TLC sheets (*i.e.* Polygram Sil G/UV254) using micropipettes. The mobile phase used was a mixture of sodium citrate and citric acid in a 2 : 1 ratio. After the development the sheets were dried and evaluated using UV light at *λ* = 254 nm and *λ* = 366 nm, iodine vapour, ninhydrin staining for detection of –NH_2_ groups, nitroprusside sodium staining for detection of –SH groups and bromocresol green for detection of –COOH groups.^40^

#### Transmission electron microscopy (TEM)

Evaluation of Absicle sizes and uniformity including analysis for polydispersity indices were carried out using TEM as described by Stollenwerk *et al.*^35^ as follows: 0.5 μl of Absicle samples were spotted onto formvar-coated copper grids. Then 5 μl of uranyl acetate (0.5 or 1%) were spotted onto the dried samples. After 30 minutes the surplus uranyl acetate was removed by use of filter paper. Digital images were acquired at 53 000 to 660 000 times magnification. In order to calculate Absicle average volume and polydispersity indices (PDI; note: in this report a perfectly uniform particle population has PDI = 1.0) the resulting images were processed in a semi-automated procedure of the Metamorph program (Zeiss, Germany), and data processed in a spreadsheet program.

#### Photon correlation spectroscopy (PCS)

Analysis of Absicle sizes and dispersities was carried out using PCS measurements as described by Stollenwerk *et al.*^35^ as follows: measurements were carried out in purified Millipore water. Absicle concentrations were chosen to produce a measurement intensity of about 300 kHz. During measurements the temperature was held constant at 23 °C. Each sample was measured for 90 minutes (3 cycles, each with 30 minutes run time). The PCS instrument was calibrated using standard 92 nm latex beads at 3-monthly intervals. Nicomp intensity weight thresholds were used at 0%, channel width was set to 10 μs, but in AUTO mode was 10 μs to 200 μs; data was collected till convergence. For data evaluation volume and intensity weighted Nicomp analysis and Nicomp software were used. Fit errors and residuals near zero signalled reliable results.

#### Zeta potential

Zeta potential of nanoparticles before and after diafiltration was measured using a ZetaSizer Nano ZSP (Malvern Instruments, Worcester-shire, U.K.). All data was analysed using the Smoluchowski relation.

#### Diafiltration

The Absicles were purified from contaminating starting molecules (*i.e.* glutaraldehyde, NaBH_3_CN) and NaCl by use of diafiltration with membranes of cut-off 100 kDa. Diafiltration was carried out as described by Abdelmoez *et al.*^47^ as follows: to ensure good purification distilled water was added to an amount 7 times larger than the volume of the original Absicle sample, additionally pressure was applied in the ranges of 10 to 20 psi. The purity of the Absicles was analysed by TLC and diafiltration was stopped as soon as no traces of contaminants remained. Diafiltration was also used as a means to exert mechanical stress on the Absicles. Their integrity after diafiltration was analysed by TEM and PCS measurements.

#### Sodium dodecylsulfate polyacrylamide gel electrophoresis (SDS-PAGE)

Evaluation of Absicle integrity and stability was carried out by use of SDS-PAGE electrophoresis as described by Abdelmoez *et al.*^47^ as follows: to perform electrophoretic analysis gels containing 7.5% Tris–HCl without SDS were used. The amount of SDS necessary to denature the proteins was present only in the sample- and running-buffers. The concentrations of the samples were adjusted to 0.5 or 1 μg μl^−1^ and were heated to 95 °C for 5 minutes. Subsequently they were loaded onto the gel together with a molecular weight standard. The loading volumes were 10 μl for the standard and 20 μl for each sample, the gel running conditions were set to 50 V for 5 minutes and 150 V for 60 minutes. After electrophoresis was complete the gel was removed, stained with Coomassie Brilliant Blue for 1 hour and destained until the desired background was reached.

#### Protein content of the Absicles

The protein content of purified Absicles was measured *via* UV spectroscopy as described by Stollenwerk *et al.*^35^ as follows: UV spectroscopy was carried out using an absorbance value of 280 nm. HSA standard solutions of 0.5 to 1.5 mg ml^−1^ were measured for calibration. The sample concentrations were determined according to these standard curves. The reliabilities of the measurements were checked by measuring standard HSA solutions of 0.5 and 1 mg ml^−1^ in parallel with each Absicle sample.

## Author contributions

GC. T: conceptualisation, methodology, validation, investigation, writing—original draft preparation; writing—review and editing; visualization. P. D: conceptualisation, methodology; validation; writing—review and editing. All authors have read and agreed to the published version of the manuscript.

## Conflicts of interest

GC. T and P. D are shareholders of Stams Diagnostics GmbH and inventors of filed patent applications in the field of uniform and reproducible albumin nanoparticles.

## Supplementary Material
